# Recent Advances in Molecular Biology of Human Bocavirus 1 and Its Applications

**DOI:** 10.3389/fmicb.2021.696604

**Published:** 2021-06-16

**Authors:** Liting Shao, Weiran Shen, Shengqi Wang, Jianming Qiu

**Affiliations:** ^1^Beijing Institute of Radiation Medicine, Beijing, China; ^2^Department of Microbiology, Molecular Genetics and Immunology, University of Kansas Medical Center, Kansas City, KS, United States

**Keywords:** human bocavirus, parvovirus, replication, gene expression, viral vector

## Abstract

Human bocavirus 1 (HBoV1) was discovered in human nasopharyngeal specimens in 2005. It is an autonomous human parvovirus and causes acute respiratory tract infections in young children. HBoV1 infects well differentiated or polarized human airway epithelial cells *in vitro*. Unique among all parvoviruses, HBoV1 expresses 6 non-structural proteins, NS1, NS1-70, NS2, NS3, NS4, and NP1, and a viral non-coding RNA (BocaSR), and three structural proteins VP1, VP2, and VP3. The BocaSR is the first identified RNA polymerase III (Pol III) transcribed viral non-coding RNA in small DNA viruses. It plays an important role in regulation of viral gene expression and a direct role in viral DNA replication in the nucleus. HBoV1 genome replication in the polarized/non-dividing airway epithelial cells depends on the DNA damage and DNA repair pathways and involves error-free Y-family DNA repair DNA polymerase (Pol) η and Pol κ. Importantly, HBoV1 is a helper virus for the replication of dependoparvovirus, adeno-associated virus (AAV), in polarized human airway epithelial cells, and HBoV1 gene products support wild-type AAV replication and recombinant AAV (rAAV) production in human embryonic kidney (HEK) 293 cells. More importantly, the HBoV1 capsid is able to pseudopackage an rAAV2 or rHBoV1 genome, producing the rAAV2/HBoV1 or rHBoV1 vector. The HBoV1 capsid based rAAV vector has a high tropism for human airway epithelia. A deeper understanding in HBoV1 replication and gene expression will help find a better way to produce the rAAV vector and to increase the efficacy of gene delivery using the rAAV2/HBoV1 or rHBoV1 vector, in particular, to human airways. This review summarizes the recent advances in gene expression and replication of HBoV1, as well as the use of HBoV1 as a parvoviral vector for gene delivery.

## Introduction

Human bocavirus 1 (HBoV1), a human parvovirus, was discovered from human nasopharyngeal specimens in 2005 (Allander et al., 2005), Parvoviruses are a non-enveloped, icosahedral virus with linear single-stranded DNA (ssDNA) genomes ranging between 4.7 and 5.7 kilobases (kb) (Cotmore et al., 2014). There are two subfamilies within the *Parvoviridae* family, *Parvovirinae* which infects vertebrates, and *Densovirinae*, which infects invertebrates. The *Parvovirinae* subfamily has eight genera, including *Amdoparvovirus, Aveparvovirus, Bocaparvovirus, Copiparvovirus, Dependoparvovirus*, *Erythro-parvovirus*, *Protoparvovirus*, and *Tetraparvovirus* (Cotmore et al., 2019). HBoV1, bovine parvovirus (BPV) isolated early in 1961 (Abinanti and Warfield, 1961), and canine minute virus (or minute virus of canines, MVC) isolated in 1967 (Binn et al., 1970), are the three representative members in the genus *Bocaparvovirus*. Recently, several new members have been identified and classified (Cotmore et al., 2019), including gorilla bocavirus (GBoV1), porcine bocavirus (PBoV), California sea lion bocavirus (CslBoV), feline bocavirus (FBoV), rat bocavirus (RBoV), bat bocaparvovirus (BtBoV), dromedary camel bocaparvovirus (DcBoV), mink bocavirus (MBoV), and rabbit bocaparvovirus (RBoV) (Cheng et al., 2010; Kapoor et al., 2010; Li et al., 2011; Lau et al., 2012, 2016, 2017; Lanave et al., 2015; Yang et al., 2016; Woo et al., 2017). However, only incomplete viral sequences were identified, and no virus was able to be isolated or cultured in cells.

The ssDNA genome of parvovirus is flanked with two short terminal hairpin structures at both ends, which are critical to viral genome replication. The secondary structure of different hairpins, a partial of which has double-stranded (ds)DNA structure, are composed of both paired and mismatched nucleotides. The termini of parvovirus are divided into two categories: heterotelomeric or homotelomeric. The termini of the former group (e.g., members in genera of *Protoparvovirus, Bocaparvovirus*, and *Amdoparvovirus*) are named left-end hairpin (LEH) and right-end hairpin (REH), which correspond to the 3′ and 5′ ends of the negative sense ssDNA viral genome, respectively. For homotelomeric parvoviruses, e.g., members in genera of *Dependoparvovirus*, *Erythroparvovirus*, and *Tetraparvovirus*, the termini are called inverted terminal repeats (ITRs). Dependoparvoviruses (e.g., adeno-associated virus, AAV) are named as such because their replication relies on a helper virus, e.g., adenovirus or herpesvirus (Schlehofer et al., 1986; Ward, 2006; Weitzman and Linden, 2011). All other known parvoviruses replicate autonomously without the need of a helper virus, and therefore are called autonomous parvoviruses.

This review summarizes the recent advances in molecular biology of HBoV1, as well as use of HBoV1 as a parvoviral vector for gene delivery and in rAAV vector production.

## Genome Organization of HBoV1

**FIGURE 1:**
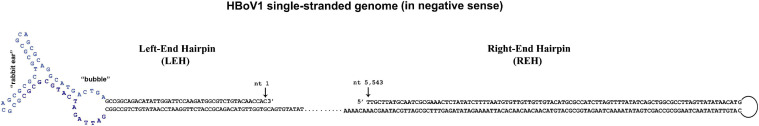
Sequence and structure of the HBoV1 left and right end hairpins. The structures of HBoV1 LEH and REH are shown with the 3′ end and 5′ end sequences, respectively, which were predicted using the DNAMAN program (Lynnon, Co., Quebec, Canada). The ear and bubble are indicated, as well as the start and end nucleotides of HBoV1 genome. The sequence refers the full-length HBoV1 genome of the isolate Salvador1 (GenBank accession no.: JQ923422).

In bocaparvoviruses, only the full-length genomes of HBoV1, BPV and MVC have been sequenced, including the terminal hairpins of both ends (Chen et al., 1986; Qiu et al., 2007; Sun et al., 2009; Huang et al., 2012). The HBoV1 genome is 5,543 nucleotides (nts) in length (Genbank accession no.: JQ923422) with distinct hairpin structures at both ends (Huang et al., 2012). The LEH is 140-nt in length and is predicted to be “Y” shaped with short axial ears and mismatches causing an unpaired bubble (Figure 1). The functions of HBoV1 LEH have not been studied. In the heterotelomeric minute virus of mice (MVM), MVM LEH plays a critical role in genome packaging and is important for transcription initiation with the help of NS1 (Li L. et al., 2013). The REH of MVC and BPV, but not HBoV1, harbor sequences that have the potential to fold into a cruciform structure near the end tip, although it is thermodynamically less favorable for BPV (Sun et al., 2009). The cruciform structure also appears in MVM, which has been proven to be required for MVM DNA replication (Astell et al., 1985; Cotmore and Tattersall, 1998, 2005b). Unique among all parvoviruses, the HBoV1 REH has a perfect palindromic sequence of 200 nts in length (Figure 1). Notably, the MVC LEH and the BPV REH were isolated both in flip and flop forms, while the other termini of these three viruses were only found in one form (Chen et al., 1986; Qiu et al., 2007; Sun et al., 2009; Huang et al., 2012). Nevertheless, we believe that DNA replication of bocaparvoviruses follows the rolling hairpin DNA replication model of other parvoviruses (Cotmore and Tattersall, 2014).

## Transcriptional Profile of HBoV1

All three bocaparvoviruses, BPV, MVC, and HBoV1, share similarities in their transcriptional expression profiles but with features different from other parvoviruses (Qiu et al., 2007; Sun et al., 2009; Chen et al., 2010). Bocaparvoviruses have one promoter with two polyadenylation sites, the proximal and distal polyadenylation sites [(pA)p and (pA)d], respectively. Therefore, bocaparvovirus only transcribes one single precursor (pre-)mRNA. This pre-mRNA undergoes alternative splicing and alternative polyadenylation to generate multiple viral mRNA transcripts. The left half of the viral genome encodes non-structural (NS) proteins, and the right half encodes structural (VP) proteins (Sun et al., 2009; Qiu et al., 2007; Chen et al., 2010; Sukhu et al., 2013). One unique feature of the bocaparvoviruses is the expression of a phosphorylated non-structural protein (NP1) (Lederman et al., 1984), whose open reading frame (ORF) is located in the middle of the genome (Qiu et al., 2007; Sun et al., 2009; Huang et al., 2012). Due to the small genome capacity, NP1 ORF largely overlaps with the NS1 ORF at the 3′ end. NP1 plays an important role in both viral pre-mRNA processing and viral DNA replication (Shen et al., 2016; Zou et al., 2016).

**FIGURE 2:**
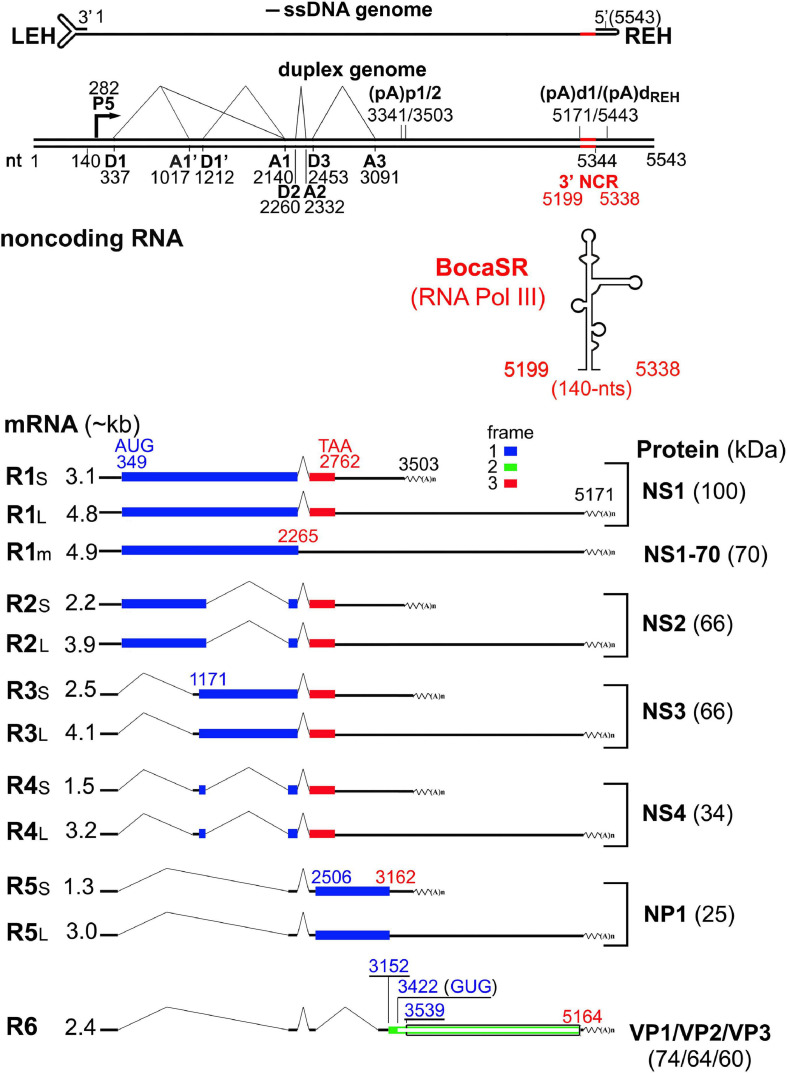
Transcription map of HBoV1. The major transcription landmarks including the terminal repeats (LEH and REH), promoter (P), splice donors (D) and acceptors (A), and (pA)p and (pA)d sites, are depicted. All identified mRNA transcripts are listed below the map (designated R1 to R6), with their respective sizes shown on the left and the detected molecular weight of the expressed proteins shown to the right. Different ORFs are illustrated in blue, red or green colors. The expressed non-coding RNA (BocaSR) is diagrammed with the size. NCR, non-coding region.

The processing of HBoV1 pre-mRNA appears to be more complex than those of MVC and BPV (Qiu et al., 2007; Sun et al., 2009; Chen et al., 2010; Zou et al., 2019; Figure 2). The mRNA spliced at the D2-A2 sites, which results in a shift of the NS1 ORF at the C-terminus, encodes NS1. Unspliced mRNA that reads through the D2-A2 intron encodes the NS1-70 protein (Chen et al., 2010). Among the NS1-coding region, alternative splicing from intron D1 to A1’, D1’-A1, and both generates R2, R3, and R4 mRNAs, respectively, which encode NS2, NS3 and NS4. R5 mRNA that is being spliced at both the D1-A1 and D2-A2 introns is responsible for NP1 expression. R6 mRNA that is consecutively spliced at all three introns, D1-A1, D2-A2, and D3-A3, encodes VP proteins. The VP2 protein is translated from a non-canonical start codon (GUG) located between VP1 and VP3, which is uncommon for parvoviruses (Janik et al., 1984; Zou et al., 2016). All NS-encoding mRNA transcripts had short (R_XS_) and long (R_XL_) forms, which are terminated at the proximal [(pA)p1 and (pA)p2] and distal polyadenylation sites [(pA)d1 and (pA)d_*REH*_], respectively (Figure 2; Hao et al., 2017; Zou et al., 2019). However, VP-encoding transcripts only use the (pA)d sites. Viral mRNA transcripts use the (pA)p1 and (pA)p2 sites at a roughly equal efficiency, but they prefer to use the (pA)d1 more than the (pA)d_*REH*_ (Zou et al., 2019).

**FIGURE 3:**
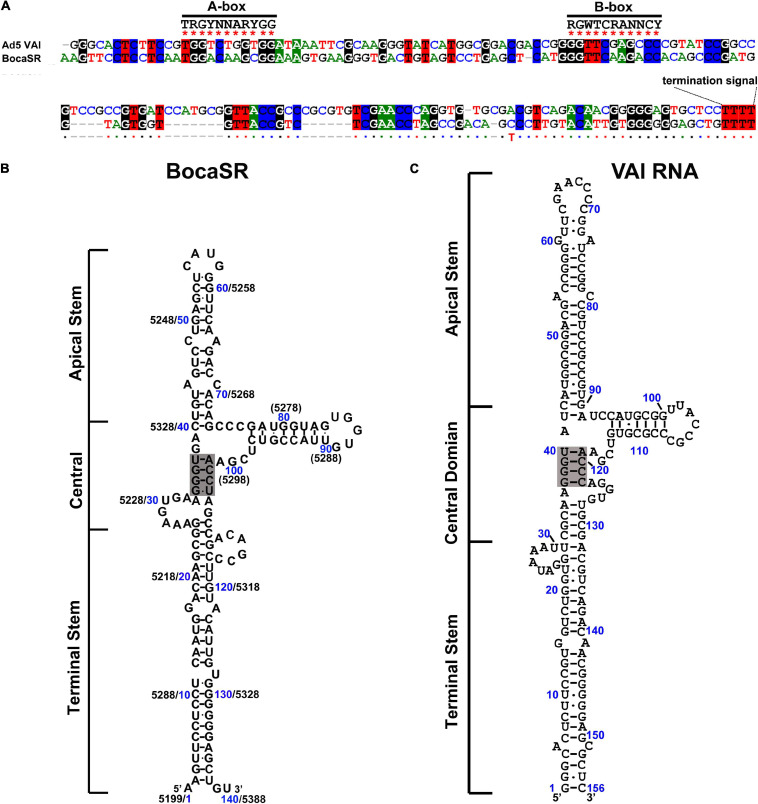
Comparisons between BocaSR and VAI RNA. **(A)** RNA sequence alignment. The sequences of BocaSR and adenovirus 5 (Ad5) VAI RNA were aligned using the CLUSTALW algorithm. Identical nucleotides are colored. Consensus sequences of the A- and B-boxes of the intergenic RNA Pol III are indicated. R represents G/A; Y, C/T, and N any nucleotides. **(B,C)** The structure of BocaSR **(B)** was predicted using the KineFold algorithm, with Ad5 VAI RNA serving as a reference **(C)**. Nucleotide numbers shown in blue and black are the positions of the BocaSR RNA (VAI RNA) sequence and the HBoV1 genome, respectively. Stem structures are indicated, and the central tetranucleotide pairs are highlighted in gray.

Unique among all the parvoviruses, HBoV1 expresses a small non-coding RNA (BocaSR) (Wang et al., 2017b), which is transcribed from nt 5,199 to 5,338 of the dsDNA genome through a RNA Pol III promoter that lies entirely within the gene and composes A- and B-boxes as those in adenovirus virus (Ad)-associated (VA) I RNA (Punga et al., 2020; Figure 3A). Its transcription level is much higher than all other HBoV1 mRNA transcripts. It is presumably folded into a secondary structure similar to VAI RNA and is composed of terminal stem, central domain, apical stem and loops (Figures 3B,C; Wang et al., 2017b).

## HBoV1 Non-Structural Proteins-NS1-4

### NS1 Protein

**FIGURE 4:**
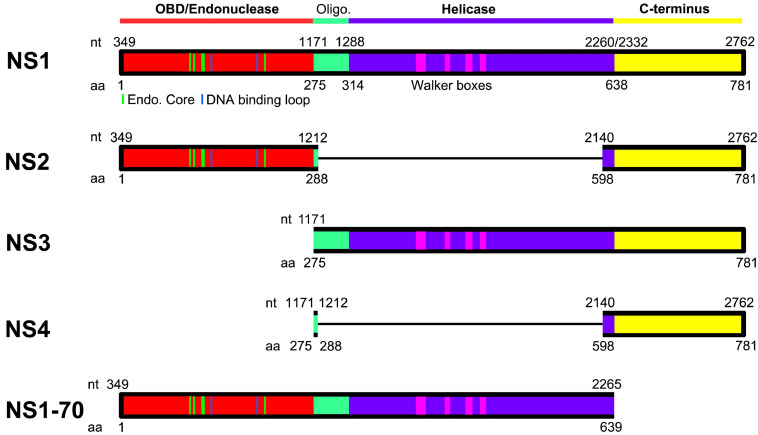
Domains of HBoV1 NS proteins. HBoV1 NS1 (GenBank: AFR53039) and AAV5 Rep78 (GenBank: AAD13755) are aligned. N-terminal origin DNA binding domain (OBD; in red) and helicase domain (in purple) are diagramed. The regions positioned between the OBD and helicase domains (shown in green) are predicted to be the oligomerization signal. The C-terminal region (shown in yellow) is predicted to serve potentially transcriptional activation function. Dashed lines in the OBD indicate residues that are structured as endonuclease core/DNA binding loop (Tewary et al., 2013), and dashed rectangles in the helicase domain indicate Walker boxes (Koonin, 1993). Oligo. indicates a putative oligomerization signal. NS2, NS3, NS4, and NS1-70 proteins are diagramed in colored blocks with thin lines indicating excised aa sequences due to ligation of the neighboring exons of their mRNAs.

The sequence similarities between BPV and HBoV1 and between MVC and HBoV1 NS1 are 31 and 39%, respectively. NS1, the largest non-structural protein of parvovirus and also named replication protein 78/68 (Rep78/68) for AAVs, plays a key role in parvovirus DNA replication (Cotmore and Tattersall, 1989; Cotmore et al., 1995). NS1 is composed of three function domains, including the N-terminal DNA-binding/endonuclease domain, the middle helicase domain, and the C-terminal transcription activation domain (Figure 4). The N-terminal domain binds to the replication origin (Ori) of viral replicative form (RF) DNA, and is referred to as the origin or DNA-binding domain (OBD/DBD). It has both strand- and sequence-specific endonuclease activity (Tewary et al., 2013). HBoV1 OBD comprises aa1–275, of which the structure has been determined (Tewary et al., 2013). Superimposition of the OBD structures of AAV5 Rep68 (Hickman et al., 2002), MVM NS1 (Tewary et al., 2015), and HBoV1 NS1 displayed a conserved beta-sheet core, flanked by several alpha helices on both the left and right sides (Tewary et al., 2013). They share similarities with each other and belong to the HUH-nuclease superfamily. One loop and an alpha helix protrusion are sequence specific and specifically bind to Ori major and minor grooves. For AAVs, the interaction between Rep78/68 OBD and the Ori has been studied in detail by analyzing the structure of the Rep78/68 and Ori complex (Hickman et al., 2004; Santosh et al., 2020). Rep78/68 oligomerizes and binds to sequence specific tetra-nucleotides repeats (Hickman et al., 2004; Santosh et al., 2020). In HBoV1, the two DNA binding regions of the HBoV1 OBD are positively charged, and the mutation of which greatly diminished viral genome replication (Shen et al., 2016). However, the HBoV1 OBD/Ori complex has not been structurally resolved.

The middle domain of the NS1 protein contains four conserved Walker motif (boxes A, B, B’, and C), which belongs to the SF3 helicase family and carries out 3′–5′ helicase function (James et al., 2003; Cotmore and Tattersall, 2005a). By alignments of HBoV1 NS1 with AAV2 Rep78 and MVM NS1 (Legendre and Rommelaere, 1994; Smith et al., 1997), the HBoV1 C-terminal domain (aa 638–781) is predicted to have transcription transactivation capability (Figure 4), but has not been studied (Legendre and Rommelaere, 1994; Smith et al., 1997).

NS1-70 is the short version of the NS1 lacking the C-terminus. Its expression level is very low during virus infection (Shen et al., 2015). But the protein *per se* can induce a DNA damage response (DDR) in a comparable capability with the full-length NS1 (Deng et al., 2016a), and thus it is supposed to support viral DNA replication.

### NS2, NS3, and NS4 Proteins

The function of HBoV1 NS2-4 has not been well studied, and is dependent on the cell types used. NS2 is required for HBoV1 infection in polarized human airway epithelia, whereas NS3 and NS4 are not required (Shen et al., 2015). Notably, all three are not required for replication of the HBoV1 duplex genome (an infectious clone) in HEK293 cells (Shen et al., 2015). NS2 is unique in all parvoviruses in that it spans the OBD and the putative transcription activation domain (Figure 4). It is required for productive AAV2 infection in both HEK293 and HeLa cells, together with NP1 and BocaSR (Wang et al., 2017a). NS3 overlaps completely with the NS1 helicase coding region (Figure 4), and thus it possibly shares similar functions as the Rep52 of AAVs. Rep52 shares the helicase and C-terminal domains with Rep78, and plays an important role in viral genome packaging (Smith and Kotin, 1998; King et al., 2001). NS4 is also unique in parvoviruses. It is the putative transcription transaction domain, which is also encompassed by all of the NS1-3 (Figure 4). It can substitute the function of NS2 in supporting replication of AAV dsDNA genome (infectious clone) in HEK293 and HeLa cells (Wang et al., 2017a). Interestingly, NS4 has only 199 aa with a predicted size of 22-kDa, however, it appeared as ∼34-kDa in SDS-polyacrylamide gene electrophoresis (Shen et al., 2015). This increased molecular weight of NS4 can explain the detected sizes of the NS1-3 which are relatively large than their coding capability. Therefore, all NS1-4 are likely post-translationally modified at the C-terminal domain (NS4).

## HBoV1 Non-Structural Protein-NP1

Conserved among all bocaparvoviruses, NP1 has ∼200 aa and is expressed from an ORF that overlaps with the C-terminus of the NS1. Although there is an identity of only ∼48% among the NP1 of different bocaparvoviruses in amino acid sequence, NP1 is conserved in functions. HBoV1 NP1 has a non-canonical nuclear localization signal located at aa 7–50 (Li Q. et al., 2013). NP1 plays an enhancement role in viral DNA replication (Sun et al., 2009). Transfection with a NP1-knockout clone of HBoV1 or MVC barely produced monomeric and dimeric RF DNA (Sun et al., 2009; Huang et al., 2012; Sukhu et al., 2013; Shen et al., 2016). The NP1 proteins of BPV and MVC are exchangeable with each other, and HBoV1 NP1 is also able to supplement the function of MVC NP1 (Sun et al., 2009). Notably, NP1 could overcome the deficiency of MVM NS2 at the early replication stage by localizing at the viral DNA replication centers, but it could not compensate for the late steps of MVM infection (Mihaylov et al., 2014).

NP1 plays multiple roles during processing viral pre-mRNA (Sukhu et al., 2013; Fasina et al., 2015, 2017; Zou et al., 2016). Both MVC and HBoV1 NP1 facilitate viral pre-mRNA to read through the (pA)p site in order to generate full-length VP-encoding transcripts (Zou et al., 2016; Fasina et al., 2017). Additionally, MVC NP1 controls the expression of MVC NS proteins via its role in governing mRNA splicing of the third intron (Fasina et al., 2017). Cellular cleavage and polyadenylation specificity factor 6 (CPSF6) interacts with both MVC and HBoV1 NP1 (Dong et al., 2019; Wang et al., 2020). CPSF6 is one of the cellular factors in the polyadenylation complex that associate with the AAUAAA motif of the polyadenylation signal (Yang et al., 2010). CPSF6 tempers MVC NP1’s suppression of the internal polyadenylation at (pA)p, enhances the splicing of the third intron, and further modulates the export of MVC mRNA (Dong et al., 2019). Notably, CPSF6 also involves nuclear import of HBoV1 NP1 (Wang et al., 2020).

**FIGURE 5:**
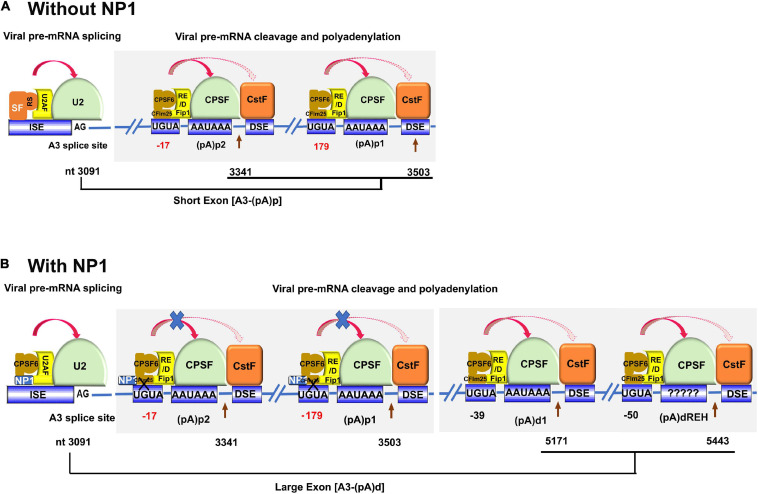
A proposed model of NP1-facilitated definition of the VP-encoding exon. The viral pre-mRNA is shown in part with the A3 splice site, (pA)p1, (pA)p2, (pA)d1, and (pA)d_*REH*_ sites. Key signals for mRNA processing, i.e., AG at the 3′ acceptor, CFIm25-binding site UGUA, CPSF-binding site AAUAAA, are shown with the downstream element (DSE) indicated. The 3′ acceptor U2 interacting complex (U2AF/U2 snRNP) and the CPSF complex composed of CFIm, Fip1, CPSF, and CstF are diagrammed. CPSF6 interacts with Fip1 through their RS and RE/D domains. Nucleotide (nt) numbers show the location of A3 3′ splice site, and the cleavage site of each pA signals. **(A)** Without NP1. There are potential SR proteins (SF) binding to the A3 acceptor to enhance the binding of U2 snRNP to the A3 acceptor. CFIm binds to UGUA enhancers at 17 (too close) and 179 nts (too far) upstream of the (pA)p2 and (pA)p1, respectively. The distance between the A3 acceptor and (pA)p sites is short, which favors defining the exon between A3 and (pA)p sites. **(B)** With NP1. Potential interaction of NP1 with CPSF6 disrupts the interaction between CPSF6 and CFIm25 that binds UGUA sites 17 (too close) and 179 nts (too far) upstream of the (pA)p2 and (pA)p1, respectively. While the CFIm25 binds UGUA signals at –39 and –50 nt (optimal distance) upstream of the (pA)d1 and (pA)d_*REH*_, respectively, the interaction between CFIm25 and CPSF6 is tight and, therefore, difficult to be interrupted by the NP1. The overall interaction between the U2 snRNP complex at the A3 acceptor and the CPSF complex at the (pA)d sites determines the large exon between the A3 acceptor and (pA)d sites.

The U2 small nuclear ribonucleoprotein (snRNP) complex deposited on the A3 acceptor is critical for the communication between the A3 acceptor and the polyadenylation site that interacts with the 3′-end CPSF complex. The interaction between the U2 snRNP and CPSF complexes defines the size of the last exon of viral mRNAs (Kyburz et al., 2006), the short exon that encodes no proteins (Figure 5A) or the large exon that encodes VP (Figure 5B). When NP1 is not presented, the short exon is preferably defined, as the distance between the last acceptor and the (pA)p signals is short, which permits a strong interaction between the U2 snRNP and CPSF complexes (Figure 5A). While NP1 is expressed, NP1 decreases the interaction of the CPSF complex on the (pA)p sites, which generates a stronger interaction of the U2 snRNP on the A3 site with the CPSF complex on the (pA)d sites; therefore, NP1 is required to define the large 3′ exon, the VP-encoding exon (Figure 5B).

The cleavage factor Im (CFIm) complex consists of a small subunit CFIm25 and two alternative large subunits, CPSF6 and CFIm59, both of which are members of the SR superfamily proteins (Ruegsegger et al., 1998). CFIm25 forms a dimer, which is bound by CPSF6 or CFIm59 via its RNA recognition motif (RRM) domain to form a tetrameric CFIm complex (Yang et al., 2011). CPSF6 has a stronger arginine–serine-rich (RS) domain than the CFIm59, and CFIm25 binds specifically to a UGUA signal of the mRNA in the proximity to the CPSF-binding hexanucleotide signal (AAUAAA) (Yang et al., 2010). CFIm is a UGUA-dependent activator that promotes mRNA 3′-processing complex assembly. CFIm activator function requires the RS-like domains of CPSF6 or CFIm59, and it involves a mechanism similar to SR protein-mediated splicing regulation. Recently, it has been reported that the function of CFIm as a UGUA enhancer-dependent activator of mRNA processing contributes to its role in regulating global alternative polyadenylation (Martin et al., 2012). The direct interaction of the NP1 with CPSF6 could disrupt the interaction between CPSF6 with CFIm25, which would decrease the loading of the CFIm complex to the UGUA site upstream of the CPSF complex and suppress the cleavage at the (pA)p sites (Figure 5B). Of note, the function of CFIm in facilitating cleavage is UGUA position dependent (Yang et al., 2010; Zhu et al., 2018). A single copy of UGUA had the highest activities at −39 nt and then at −50 nt from the cleavage site (Zhu et al., 2018). We hypothesize that the CFIm binding to the two UGUA enhancers upstream of the two (pA)p sites, which are at suboptimal distances (−17 and −179 nt), is weaker than the CFIm binding to the enhancers (−39 and −50 nt) in front of the (pA)d sites (Figure 5B). Therefore, NP1 competes to interact with CPSF6, which likely disrupts the CFIm complex at the (pA)p sites but not the one at the (pA)d sites.

## HBoV1 Encodes a Small Non-Coding RNA-Bocasr

BocaSR is essential for HBoV1 replication in infected polarized human airway epithelial cultures and viral DNA replication in viral duplex genome-transfected HEK293 cells (Wang et al., 2017b). BocaSR is localized in the nucleus. It shares a high similarity of 46.1–51.2% with the other four RNA Pol III-transcribed viral small RNAs: VAI, VAII, EBER1, and EBER2. BocaSR regulates the expression of NS1, NS2, NS3, and NP1 but not NS4. However, unlike VA RNAs which are localized in the cytoplasm (Vachon and Conn, 2016), BocaSR does not inhibit phosphorylation of protein kinase R (PKR) and eukaryotic initiation factor 2 (eIF-2) (Wang et al., 2017b).

In addition to the function in enhancing viral NS protein expression, BocaSR plays a direct role in viral DNA replication, which cannot be fully complemented by VAI RNA (Wang et al., 2017b). The mechanism of how BocaSR facilitates viral genome replication has not been revealed. Intriguingly, alignments of HBoV2-4 and GBoV sequences, but not other members in the genus of *Bocaparvovirus*, show that they also express BocaSR, and at least HBoV3 BocaSR could supplement the lack of BocaSR in NS expression of HBoV1 (Wang et al., 2017b). Importantly, in the case of HBoV1 helped AAV replication, BocaSR is essential to help AAV replication in human airway epithelia, HEK293 and HeLa cells (Wang et al., 2017a). Considering the specific tissue tropism of HBoVs, this short viral non-coding RNA may evolve to contribution of bocaparvovirus adaptation to the airway or gastrointestinal environment.

## Structural Proteins

Different from BPV and MVC, HBoV1 expresses three VP proteins, and the VP2 uses a non-canonical start codon (Zou et al., 2016). The capsid proteins are expressed in a ratio ∼1:1:10 during infection, which is similar to that of AAVs. VP3, the abundant structure protein, is able to assemble virus like particles (VLPs), which contain neutralizing epitopes and receptor binding sites (Gurda et al., 2010). A monoclonal antibody 15C6 reacts with all of the VLPs formed by HBoV1-4 (Gurda et al., 2010). HBoV1 has a short VP1 unique region (VP1u) of 90 aa and its coding sequence contains internal polyadenylation signals (Zou et al., 2016). Most members in the *Parvovirinae* subfamily, except Aleutian mink disease virus (AMDV) (Huang et al., 2014) and the recently identified shrimp densovirus (Pénzes et al., 2020), contain a phospholipase A2 (PLA_2_) domain within VP1u (Zadori et al., 2001). The HBoV1 VP1u region exhibits a PLA_2_ activity within the region 11–66 aa (Qu et al., 2008). The PLA_2_ domain of the VP1u was also confirmed in MVC (Sun et al., 2009). The phospholipase activity of the PLA_2_ domain is supposed to be important for sequential structural changes from receptor mediated endocytosis to endosome escape (Zadori et al., 2001; Farr et al., 2005).

Conserved with the structures of other parvoviruses (Mietzsch et al., 2019). HBoV1-4 share the icosahedral fivefold axis tunnel, threefold axis trimeric protrusions and a twofold axis depression (Gurda et al., 2010; Kailasan et al., 2016; Mietzsch et al., 2017; Luo et al., 2021). Compared with AAVs, HBoV1 has a relative flat capsid shell (Kailasan et al., 2016). This feature makes it to be able to package a larger viral genome with an even smaller VP3. The surface variable region (VR) III of VP3 has been proposed as a host tissue-tropism determinant, which is structurally similar among the gastrointestinal tropic HBoV2-4, but different from the airway tropic HBoV1. Six monoclonal antibody epitopes on the HBoV1-4 have been determined (Kailasan et al., 2016). These characterizations of the HBoV capsid surface structures are important for development of HBoV-based viral vectors.

## HBoV1 DNA Replication

Replication of the parvovirus genome is greatly associated with DNA damage and repair factors (Schwartz et al., 2009; Adeyemi et al., 2010; Lou et al., 2012; Vogel et al., 2012; Cotmore and Tattersall, 2013; Deng et al., 2016b). Upon infection, H2AX and RPA32 are phosphorylated, and the signals are passed through phosphoinositide 3-kinases ATM (Ataxia telangiectasia mutated), ATR (ATM- and RAD3-related), and DNA-PKcs (DNA-dependent protein kinase catalytic subunit), respectively. Activation of the phosphoinositide 3-kinases causes cell cycle arrest and activates DNA repair pathways. However, HBoV1 infects polarized human airway epithelia, which are terminally differentiated (mitotically quiescent). HBoV1 infection activates ATM, ATR and DNA-PK, which play significant roles in viral DNA replication (Deng et al., 2016b). All three DDR pathways were activated during HBoV1 duplex genome transfection of HEK293 cells and are necessary for viral DNA replication (Deng et al., 2016a). NS1 alone induces DDR signaling but no cellular DNA damage. Importantly, DNA repair polymerase (Pol) κ and Pol η play a significant role in HBoV1 genome replication in both polarized human airway epithelia and HEK293 cells (Deng et al., 2016a,b).

**FIGURE 6:**
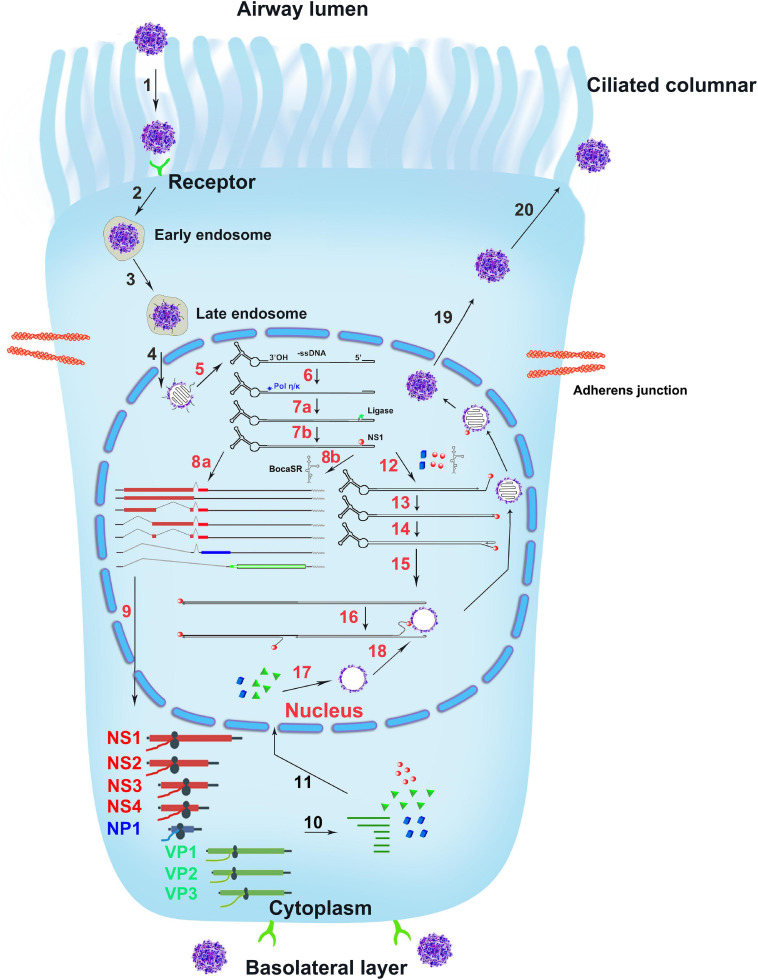
The infection life cycle of HBoV1. A ciliated airway epithelial cell is depicted with diagrams of the cilia and junction molecules. HBoV1 enters the cells through binding to an unknown viral receptor, which is expressed on both the apical (ciliated) and the basal cells as indicated, and through receptor-mediated endocytosis, followed by intracellular trafficking (Steps 1–3). The virus escapes from the late endosome and enters the nucleus (Step 4). In the nucleus, the uncoated ssDNA viral genome is converted to replicative form dsDNA that expresses viral NS proteins and BocaSR (Steps 5–8). The viral DNA further replicates in the nucleus (Steps 12–16) and expresses both viral NS and capsid proteins (Steps 9–11), followed by genome packaging into empty capsid (Steps 16–18). Lastly, the matured virus egresses out of the infected cells (Steps 19, 20). The HBoV1 infection cycle in the ciliated epithelial cell is illustrated based on the studies on HBoV1 and references from other parvoviruses, which are explained in the text.

HBoV1 DNA replication follows a model of DNA repair (Deng et al., 2016a,b), in contrast to the DNA replication DNA polymerase-dependent replication model of other parvoviruses (Astell et al., 1983, 1985; Berns, 1990; Ryan et al., 1996; Cotmore et al., 2000; Cotmore and Tattersall, 2005b; Ward, 2006). Upon entering the nucleus, the viral genome is recognized by Pol κ, Pol η, or both that synthesize the complementary strand, primed by the 3′-OH at the LEH (Figure 6, Steps 6, 7). In addition, for HBoV1 replication, NS1, NP1 and BocaSR are required (Figure 6, Step 12). They are localized within the viral DNA replication centers. During MVM replication, cellular factors bind both LEH and REH and are necessary for the MVM DNA replication (Christensen et al., 1997a,b; Cotmore and Tattersall, 1998). For the replication of HBoV1, such REH or LEH-binding cellular factors should be required for viral DNA replication as well but have not been identified yet.

The minimal Ori of the HBoV1 REH has been identified from nt 5357 to 5402 (Shen et al., 2016). It contains a nicking site and 4 repeats of TGT that are likely NS1-binding elements. In members of the *Parvovirinae* subfamily, the NS1- or Rep78/68-binding elements harbor tetra-nucleotides repeats (Weitzman et al., 1994; Cotmore et al., 1995; Lorson et al., 1996; Stracker et al., 2004; Tewary et al., 2014). MVM has cognate or degenerated NS1-binding sites throughout its genome (Christensen et al., 1995). However, densoviruses *Galleria mellonella densovirus* (GmDNV) and *Junonia coenia densovirus* (JcDNV) contain an NS1-binding site of trimer nucleotides repeated four times (Ding et al., 2002; Tijssen et al., 2003). The HBoV1 NS1 binds four TGT repeats located in the REH and nicks the Ori before a “T” which is located 12 bp upstream of the NS1-binding elements (Shen et al., 2016). Thus, HBoV1 NS1-binding elements share more similarities with that of the densoviruses than other bocaparvoviruses. Intriguingly, no similar NS1-binding elements are presented on the HBoV1 LEH.

## HBoV1 Life Cycle

The duplex HBoV1 genome (pIHBoV1, the infectious DNA clone) replicates in HEK293 cells, and produces progeny virions at a high titer by transfection (Huang et al., 2012). Notably, HEK293 cells do not permit HBoV1 infection. The purified virions are infectious to polarized primary human airway epithelia (Dijkman et al., 2009; Huang et al., 2012; Yan et al., 2020), which mimics virus infection of the natural host, the human airway epithelia1. HBoV1 directly infects primary human airway epithelia cultured at an air-liquid interface (HAE-ALI) through both the apical and the basolateral surfaces, indicating that the virus receptor is expressed on both the apical (ciliated) and the basal cells (Figure 6, Steps 1, 2). Parvoviruses in general enter the cells through receptor-mediated endocytosis (Step 2) (Bartlett et al., 2000; Ros et al., 2002; Parrish, 2010). The receptor for HBoV1 entry is currently unknown. After entry, HBoV1 is likely trafficked through the early to late endosomes (Step 3) (Vihinen-Ranta et al., 1998; Sanlioglu et al., 2000). Within the nucleus, the viral genome is released and recognized by the cellular DNA damage and repair machinery (Steps 5, 6). Then, the complementary strand of the viral ssDNA genome is synthesized, transcribed (Step 7), and followed by expression of viral NS proteins. NS proteins are associated with the viral genome during the replication steps (Steps 7, 12–16) and are required for genome packaging (Steps 16–18) (Chejanovsky and Carter, 1989; Dubielzig et al., 1999; Sonntag et al., 2011). The dsDNA templates undergo transcription, protein and BocaSR expression and DNA replication (Steps 8–10, 12–16). Capsid proteins produced in the cytoplasm are assembled into oligomers before translocating into the nucleus (Step 11) (Lombardo et al., 2000). Capsids are assembled in the nucleus (Steps 17–18) (Wistuba et al., 1997; Hoque et al., 1999). Eventually, the mature virions are released from the nucleus into the cytoplasm and are then transported outside of the infected cell (Steps 19–20). Apparently, much of the HBoV1 life cycle awaits being experimentally demonstration.

## HBoV1 Is a Novel Helper for AAV2 Replication in Human Airway Epithelia

HBoV1 has been demonstrated to facilitate productive AAV2 infection in polarized human airway epithelia (Wang et al., 2017a). In both HEK293 and HeLa cells, the transfected HBoV1 duplex genome (pIHBoV1) rescues the AAV2 duplex genome replication at an efficiency similar to that from the adenovirus helper genes-expressing plasmid (pHelper). The minimal essential HBoV1 units that facilitate AAV2 DNA replication and virus production are NP1, BocaSR, and NS4 genes in AAV2 duplex genome (an infectious clone)-transfected HEK293 and HeLa cells (Wang et al., 2017a). However, during AAV2 infection of HEK293 and HeLa cells, NS2 is required for AAV2 DNA replication and progeny production. Compared with Ad pHelper, the poor transactivation of the P19 and P40 promoters by AAV2 Rep78/68 in the presence of HBoV1 helper gene expression prevents the HBoV1 helper in becoming a competitive choice in rAAV vector production in HEK293 cells. Notably, expression of the HBoV1 genes (NP, NS2, and BocaSR) in HeLa cells can fully rescue replication of a full-length AAV2 clone, whereas expression of Ad E2, E4of6 does not (Wang et al., 2017a). We speculate that the presence of the ITR in front of the P5 promoter likely facilitates the expression of Rep78/68, which transactivates the downstream P19 and P40 promoters (Qiu and Pintel, 2002). Importantly, when HBoV1 NP1 and NS2 genes are combined with Ad helper genes in a plasmid pABHelper, their expression increases the rAAV2 genome replication and significantly enhances rAAV2 vector production by more than twofold (Wang et al., 2018). Thus, the pABHelper is a novel synergistic helper plasmid for rAAV vector production.

## HBoV1 Capsid-Based rAAV Vectors

The capsid of one parvovirus is able to pseudopackage the genome from another parvovirus. Previous studies succeeded in producing the chimeric parvovirus AAV2/B19, in which parvovirus B19 capsid was used to pseudopackage the AAV2 genome (Ponnazhagan et al., 1998). However, the cross genera packaging efficiency remained poor, and the rAAV2/B19 vector is not practically employed in gene therapy (Fakhiri and Grimm, 2021). The rAAV2 genome can also be packaged by the HBoV1 capsid to assemble a chimeric parvoviral vector, rAAV2/HBoV1. Strikingly, the HBoV1 capsid can package an oversized rAAV genome up to 5.8 kb without sacrificing the packaging efficiency. By carrying a full-length CFTR cDNA of 4.5-kb, it could rescue approximately one third of the cystic fibrosis transmembrane conductance regulator (CFTR) function in the CF phenotype of human airway epithelia (Yan et al., 2013). Importantly, the rAAV2/HBoV1 vector is capable of efficiently transducing the lungs of both newborn and juvenile ferrets but predominantly in the distal airways, supporting that the rAAV2/HBoV1 vector can be used for preclinical development of lung gene therapy in cystic fibrosis using ferret models (Yan et al., 2017). In addition, the HBoV1 capsid can package an rHBoV1 genome as a viral vector (Yan et al., 2013). These properties of HBoV1-based vectors provide a new tool for airway gene delivery applications.

**FIGURE 7:**
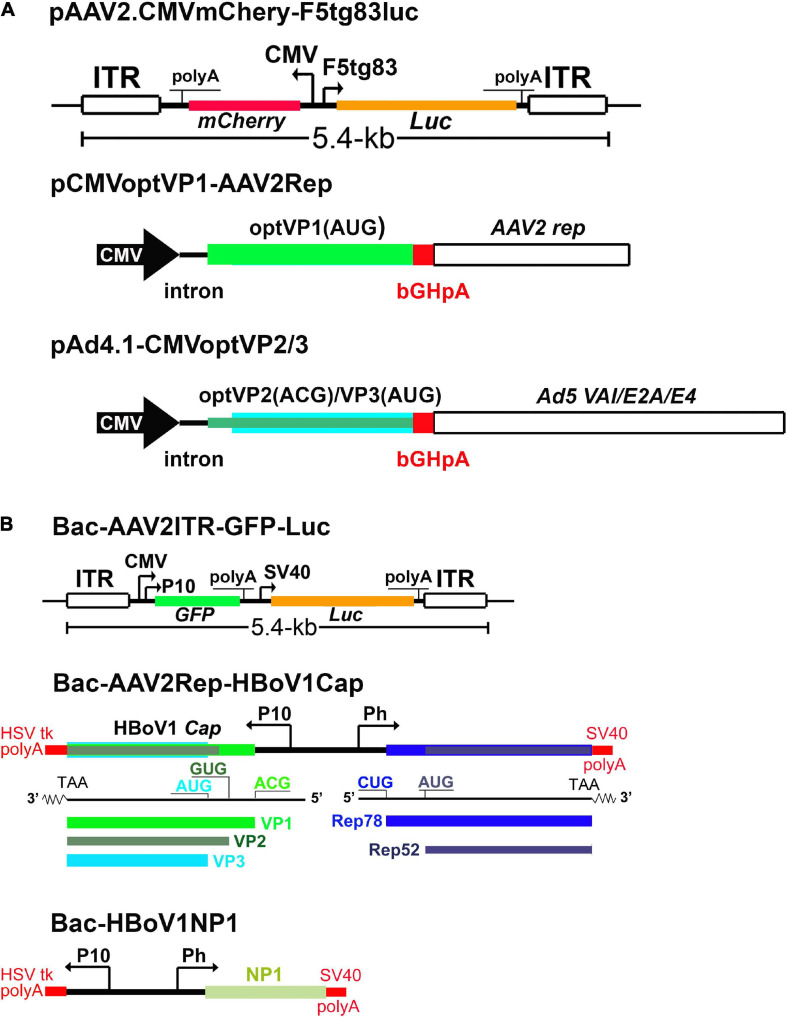
rAAV2/HBoV1 vector production systems. **(A)** NS-free rAAV2/HBoV1 vector production system in HEK293 cells. pAAV2 carries the genes of interest in rAAV2 genome. pCMVoptVP1-AAV2Rep is a two-in-one plasmid, in which the VP1 and Rep2 (Rep78/52) are expressed in two independent cassettes. pAd4.1-CMVoptVP2/3 has the optimized (opt)VP2/3 expression cassette in the Ad helper gene expression plasmid pAd4.1. **(B)** rAAV2/HBoV1 baculovirus expression vector (BEV) production system. Bac-AAV2ITR-GFP-Luc carries an rAAV2 genome. Bac-AAV2Rep-HBoV1Cap expresses AAV2 Rep proteins and HBoV1 capsid proteins. Bac-HBoV1NP1 expresses HBoV1 NP1. P10 and Ph are baculoviral promoters, and CMV and F5 tg83 are a human cytomegalovirus major immediate-early promoter and a synthetic promoter, respectively. Various polyadenylation signals are indicated as polyA. *Luc*: firefly luciferase gene; *GFP*, enhanced green fluorescent protein gene, and *mCherry*, a monomeric red fluorescent protein gene. They can be replaced by any gene of interest (GOI) for expression in the vector.

A high yield production system of rAAV2/HBoV1 has been established in HEK293 cells, which is independent on any NS proteins (Yan et al., 2019). This NS-free vector production system uses co-transfection of 3 plasmids in HEK293 cells, including one trans helper plasmid encoding both HBoV1 VP1 and the AAV2 Rep proteins, and another encoding VP2, VP3 and Ad helper genes (Figure 7A). This system yielded > 16-fold more vectors than the prototype 4-plasmids system (Yan et al., 2013), while retaining the same transduction activity. In addition, the HBoV1 capsid can pseudopackage an rAAV2 genome in insect Sf9 cells with baculovirus help (Deng et al., 2020; Figure 7B), in which NP1 plays an enhancement role in increasing the yield of the rAAV2/HBoV1 vector. While the transduction efficiency of the rAAV2/HBoV1 vector produced in Sf9 cells is still 5∼7 times lower than that of the vector produced from HEK293 cells, the Sf9 based vector production system generated more empty particles (accounting for ∼50%), which is a barrier to large quantity vector production in Sf9 cells. Nevertheless, as the Sf9 cell culture can be easily scaled up in a bioreactor, the Sf9-based rAAV2/HBoV1 vector system holds promise to produce the vector in a large quantity.

Four additional bocaparvovirus vectors, HBoV2, HBoV3, HBoV4, and GBoV1 capsid-packaged rAAV2 genome vectors have been developed and assessed for transduction in human airway epithelial cultures and lung organoids (Fakhiri et al., 2019). While rAAV2/HBoV1, rAAV2/HBoV2 and rAAV2/GBoV vectors successfully transduced human airway epithelial cultures and lung organoids to different extents, rAAV2/HBoV4 more preferred to transduce non-ciliated cells, which are the basal/stem cells of human airway epithelia. All these features will provide more possibilities in gene therapy with exhibition of their respective strengths.

## Discussion

Since the discovery of HBoV1 in 2005, our knowledge in understanding the biology of HBoV1 has been deepened year by year, which opens a new venue to study parvovirus and facilitate development of novel parvoviral vectors for human gene therapy. The properties of HBoV1 infection and replication in human airway epithelia confer unique features to HBoV1, including its replication dependence on DNA damage and repair machinery, expression of a RNA Pol III-transcribed viral non-coding RNA, and as a *bona fide* helper for AAV replication in airway epithelia, The finding of the high efficiency in parvovirus cross-genera pseudopackaging was a surprise, which contributes an airway tropic parvoviral vector for airway gene delivery, in particular, of cystic fibrosis, as it has a packaging capability of 5.8 kb. But these are not the end, and much will be learned from HBoV1 in the future, which will further enrich our knowledge in parvoviral gene expression and DNA replication, as well as in parvoviral vector development.

## Author Contributions

LS and JQ wrote the manuscript. WS and SW revised the manuscript. All authors contributed to and approved the submitted version.

## Conflict of Interest

The authors declare that the research was conducted in the absence of any commercial or financial relationships that could be construed as a potential conflict of interest.
